# Integrative Lighting Aimed at Patients with Psychiatric and Neurological Disorders

**DOI:** 10.3390/clockssleep5040052

**Published:** 2023-12-15

**Authors:** Xinxi Zeng, Thierry Silvio Claude Soreze, Martin Ballegaard, Paul Michael Petersen

**Affiliations:** 1 Department of Electrical and Photonics Engineering, Technical University of Denmark, 2800 Kongens Lyngby, Denmark; xinze@dtu.dk (X.Z.); pape@dtu.dk (P.M.P.); 2 Department of Neurology, Copenhagen University Hospital—Zealand University Hospital Roskilde, 4000 Roskilde, Denmark; mbag@regionsjaelland.dk; 3 Department of Clinical Medicine, University of Copenhagen, 2200 Copenhagen, Denmark

**Keywords:** CIE S026, circadian, MEDI, integrative lighting, MLIT, TAT, melatonin suppression

## Abstract

The purpose of this paper is to investigate the impact of circadian lighting-induced melatonin suppression on patients with psychiatric and neurological disorders in hospital wards by using an ad-hoc metrology framework and the subsequent metrics formalized by the CIE in 2018. A measurement scheme was conducted in hospital ward rooms in the Department of Neurology, Zealand University Hospital, at Roskilde in Denmark, to evaluate the photometric and colorimetric characteristics of the lighting system, as well as its influence on the circadian rhythm of the occupants. The measurement scheme included point measurements and data logging, using a spectrophotometer mounted on a tripod with adjustable height to assess the newly installed circadian lighting system. The measured spectra were uploaded to the Luox platform to calculate illuminance, CCT, MEDI, etc., in accordance with the CIE S026 standard. Furthermore, the MLIT based on MEDI data logging results was calculated. In addition to CIE S026, we have investigated the usefulness of melatonin suppression models for the assessment of circadian performance regarding measured light. From the results, the lighting conditions in the patient room for both minimal and abundant daylight access were evaluated and compared; we found that access to daylight is essential for both illumination and circadian entrainment. It can be concluded that the measurement scheme, together with the use of the Luox platform and Canva template, is suitable for the accurate and satisfactory measurement of integrative lighting that aligns with CIE requirements and recommendations.

## 1. Introduction

Light is crucial for any indoor environment and for obtaining a good indoor climate. First of all, light enables the visual perception of our surroundings, as image-forming (IF) is used for its characterization. Secondly, the non-image-forming (NIF) effects of light anchor us to the 24 h day via a circadian timing system [1] that controls many aspects of our physiology, metabolism, and behavior, such as body core temperature, hormones, alertness, and performance [2,3,4]. Circadian rhythms are spontaneously generated and have a cycle of approximately 24 h. Yet, for the circadian system to get synchronized to the external 24 h day, it requires a robust and predictable light/dark pattern [5,6,7].

Historically, for an outdoor lifestyle, this has not been an issue; however, as we spend most of our time indoors in modern times, it is not a guarantee that we will be exposed to sufficient light [8]. Under such circumstances, the circadian timing system gets de-synchronized (free-running), which results in the same physiological response as experiencing jet lag [7,9,10]. Furthermore, improper light exposure can lead to a disrupted circadian system, which, in turn, has been strongly correlated with poor mental health, cognitive and neurological disorders, and several diseases [11,12,13]. Therefore, ensuring a proper circadian balance through lighting is critical in the context of neurological and psychiatric hospitalisation. Thus, monitoring the compliance of a given installed electrical lighting system in hospitals allows for a thorough assessment of any potential light-induced adverse effects for the patients.

In addition to the circadian timing system, light reaching the eye can also change the size of the pupil [1] and reduce night-time melatonin levels (dark hormone and perception of darkness), with high levels at night, low levels during the day [10,14,15,16]. As a consequence, alertness, performance, and mood are impacted. This alerting response is why light in the evening can make it difficult to fall asleep.

The physiological effect of light also depends on personal characteristics, such as age, light history (how much time was spent in a dimly lit room or outside early in the day), and chronotype. Ultimately, the mismatch between environmental time and physiology leads to circadian rhythm disruption and sleep problems [17,18,19,20].

The human retina comprises five types of photo-receptors, including three types of cones, usually coined after their spectral peak, which allow for color vision; these are mainly active in bright light conditions, and rods (retinal cells) are active in dim and dark lighting conditions, enabling “night vision”. These photo-receptors enable photo-transduction, which mediates both IF and NIF effects [21].

**Figure 1:**
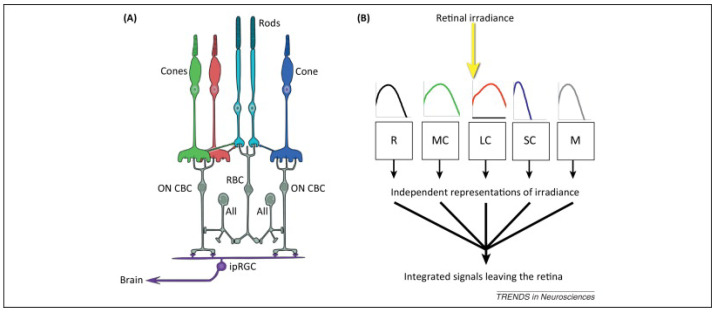
All retinal photo-receptor classes are upstream of circadian, neuroendocrine, and neurobehavioral responses to light [21]. (**A**) Photoreceptors (coloured cells) and their connection to retinal interneurons (grey cells). (**B**) Flow stream of light processing by retinal photoreceptors, and their respective spectral sensitivity. The three cones, and corresponding spectral sensitivities, are coloured according to their peak spectral sensitivity, i.e., red, blue and green.

The last type of photo-receptors is a specific class of retinal ganglion cells, termed intrinsically photo-sensitive retinal ganglion cells (ipRGCs); these can directly sense light through their photo-sensitive pigment, melanopsin (OPN4). All five types of photo-receptors are entangled and, therefore, all participate in both vision and circadian signaling. The photo-receptors and their respective spectral sensitivity to light can be seen in Figure 1 [21]. However, melanopsin signaling correlates the most with circadian-related changes, emphasizing the importance of the accurate measurement and assessment of the contribution of melanopsin-based ipRGCs [22].

With the publication of CIE S026/E:2018 CIE System for Metrology of Optical Radiation for ipRGC-Influenced Responses to Light [23], a system for metrology of optical radiation was defined for light-induced responses that can be elicited by ipRGCs (ipRGC-influenced light (IIL) responses), which should be used for quantitative and qualitative field measurements. In the second edition of the CIE International Lighting Vocabulary CIE S 017/E:2020, “integrative lighting” is the official term for lighting that is specifically intended to integrate visual and nonvisual effects, producing physiological and psychological effects on humans, as reflected by scientific evidence [24].

The CIE also published an open-access α-opic toolbox that calculates all the quantities and ratios of the α-opic metrology in the photometric, radiometric, and photon systems based on either a measured (user-defined) spectrum or selected illuminants (A, D65, E, FL11, and LED-B3) built into the toolbox [25]. The metrology comprises five α-opic irradiances and five α-opic equivalent daylight illuminances (α-opic EDIs, weighted against D65 standardized daylight light source [26]) that have a direct linear relationship with the luminous and/or radiant flux of a light source [25,27], whereby melanopsin, the photo-pigment in the ipRGC photo-receptors, is specified as “melanopic” [28]. Since melanopsin is the primary opsin-based photopigment considered from the CIE S026 framework in this analysis [23], melanopic-EDI (MEDI, lux) has been recommended as the parameter to use to describe the spectral sensitivity of nonvisual responses to light under most, practically relevant situations. Measurements should be taken at the position of the user’s eye (with a detector orientation that corresponds to the dominant direction of gaze) [27].

Furthermore, Brown et al. 2020 [22] reported daytime light recommendations for indoor environments throughout the 24 h day.

- Daytime: the recommended minimum MEDI is 250 lux at the eye measured in the vertical plane at ∼1.2 m height (i.e., vertical illuminance at eye level when seated), noting that, if available, daylight should be used in the first instance to meet these levels;
- Evening: this covers the light recommendations for residential and other indoor environments during the evening, starting at least 3 h before bedtime; the recommended maximum MEDI is 10 lux measured at the eye level in the vertical plane ∼1.2 m height. In order to help achieve this, where possible, white light should have a spectrum depleted in short wavelengths close to the peak of the melanopic action spectrum;
- Nighttime: the light recommendations for the sleep environment. The sleeping environment should be as dark as possible. The recommended maximum ambient MEDI is 1 lux measured at the position of the eye;
- For unavoidable activities where vision is required during the nighttime, the recommended maximum MEDI is 10 lux measured at the eye in the vertical plane at ∼1.2 m height.

These MEDI-based recommendations offer a powerful and straightforward framework for light researchers, designers, and other indoor professionals to have a more thorough insight into light and lighting that optimally supports human health [27].

It should be noted that the above-mentioned recommendations have not been age correlated; thus, it is the minimum requirement suitable for people of a young age [23]. The phase and amplitude of several circadian functions have been found to be altered by the aging process [29,30]. This could be partly caused by age-related alterations to the circadian system via the eye and the retina [31,32]. These age-related changes are often described in the eye, including optical or neural features that can lead to a reduction in retinal sensitivity to light [33,34]; thus, higher light intensities and probably spectral changes may be needed to support visual and nonvisual needs [35,36]. In our scheme, we have adopted the MEDI recommendations with age corrections proposed by the Good Light Group (GLG), as shown in Table 1 below [35,36].

## 2. Results

### 2.1. Measurement Scheme

The measurement scheme constitutes a combination of two measurement methods: point measurement and data logging measurement.

For point measurement, the points are selected based on the usage of the space and the tasks or roles of the occupants. For instance, in an office space, the viewing position would be that of a desk setup, with the direction of view being selected accordingly. At hospitals or care facilities, the measurement points should be set at the level of the patient’s bed at several positions, corresponding to resting or sitting positions. Several viewing conditions could also be included in the measurements. It is also critical to include measurement points according to different users. For instance, in care facilities, caregivers and patients have different roles, tasks, etc.; therefore, the rooms and facilities meant for caregivers should not be excluded during a measurement campaign at a care facility.

Apart from the points mentioned above, for data logging measurements, the “room’s representative” location (for instance, at the center of the room with the cardinal directions as the viewing directions or near a patient’s bed in a hospital setting) could also be measured continuously over a certain period of time, which may be of importance with regard to reporting light history, zeitgeber strength, temporal dynamics, and seasonality.

In our case study, both the point measurement and data logging were chosen to take place in room 9 on the first floor, building 60, Zealand University Hospital, at Roskilde, as it was equipped with a spectrally optimized “circadian” LED lighting system (designed by Chromaviso A/S, Aarhus, Denmark), which can adjust the light output and spectrum automatically.

### 2.2. Key Parameters

The following is the explanation for the terms presented in the measurement results and report:

- Illuminance (lux): a measure of how much the incident light illuminates a given surface. It can be seen as how “bright” the reflected light is perceived by the human eye.
  The value should be high enough to provide enough lighting for people to see things clearly but not too high, as this causes discomfort glare. Depending on the work environment, the requirements can be found in DS/EN12464-1:2021 [37]. In addition, GLG has published its age-correlated recommendations for illuminance levels [36].
- MEDI (lux): this describes the response of the nonvisual photo-receptors, i.e., ipRGCs, in the human eye in correspondence to a standardized D65 illuminant. This response is indicative of the photo-biological effect of a given illumination condition on the circadian system of the exposed subjects and is a combination of the spectrum of light and intensity. It provides an indication of the ability of a light stimulus to entrain the circadian system as well as suppress melatonin in the blood. A high MEDI during the day is usually supportive of alertness, the circadian rhythm, and a good night’s sleep. At night-time, a low MEDI promotes sleep [23].
- Melanopic daylight efficacy ratio (MDER): a spectral metric of the biological effect of an artificial light source compared to daylight (6500 K), which is then divided by the photopic response (ratio) to estimate the nonvisual light. The ratio provides a shorthand to estimate the relative nonvisual stimulus of a light source while maintaining visual standards. As a rule of thumb, a higher ratio will have a higher melanopic content (a DER bigger than 0.8 represents stimulating light), and a lower ratio represents lower melanopic content (a DER lower than 0.3 represents sleep-promoting light). Typically, artificial lighting has a lower biological effect than daylight, with the MDER being below 1 [23].
- Correlated color temperature (CCT, K): a visual measure to describe the colored appearance provided by a white light source perceived by the human eye. CCT is based on the temperature in kelvin needed to warm a blackbody to achieve the color appearance. A range of 2700–3000 K is called ‘warm color’, and a CCT above 5000 K is called ‘cool color’.
- Color rendering index (CRI, Ra): the ability of a light source to render the colors of various objects faithfully in comparison with an ideal or natural light source. The higher the CRI, the more accurate the color rendering of a given light source is, with the maximum achievable value being 100.

Apart from the above-mentioned parameters, which are obtained from both point measurement and data logging, the time above threshold (TAT) and mean light time (MLIT) above threshold are unique to the data logging results, as proposed by Reid et al. [38] and adapted by our group. However, in our study, we used the MEDI value instead of the illuminance value, as was the case in the work of Reid et al. [38], to calculate the TAT and MLIT; thus, we obtained insight into how long the MEDI value was over or under a specific threshold during a given selected 24 h day. This is also of importance when evaluating the strength of zeitgeber and circadian entrainment.

Further, MLIT gives insight into MEDI progression over time and the average timing of light exposure. This information might be of importance in regard to light timing, which might be of importance in regard to, e.g., phase-shifting of the circadian system and its alignment.

TAT and MLIT may be calculated for the daytime (in this study, 7 a.m.–7 p.m.) and the night-time (in this study, 11 p.m.–7 a.m.). TAT is defined as the number of time epochs (e.g., in this study, 15 min epochs) above a given threshold multiplied by 15 min.

Moreover, an MLIT above the threshold integrates information on the intensity (MEDI threshold), duration (number of 15 min epochs above the threshold), and timing (clock time of each 15 min epoch above the threshold) of light exposure.

For 24 h data logging with a 15 min measurement interval, as was the case in this study, the $MLIT^{C}$ is formulated with the general threshold C: lux for MEDI.

(1) $$MLIT^{C}=\frac{\sum_{j=1}^{96}\sum_{k=1}^{n}<I_{jk}^{C}\times Y_{j}>}{\sum_{j=1}^{96}\sum_{k=1}^{n}I_{jk}^{C}}$$

where $Y_{j}$ is the *j*th epochs and is 1 if MEDI > C on the *k*th day, with indicators: *j* = 1, …, 96; *k* = 1, …, *n* (in this study, *n* = 1 as the data logging lasted for 1 day), and *C* = 250 lux in this case. Here, *j* reaches 96 because the light exposure (in lux) is measured every 15 min for 24 h. Thus, for example, an $MLIT^{250}$ of 720 min indicates that a light exposure greater than 250 lux is, on average, centered around 720 min (or around 12 p.m. if the period starts at 12 a.m.) requirements.

### 2.3. Point Measurement

**Figure 2:**
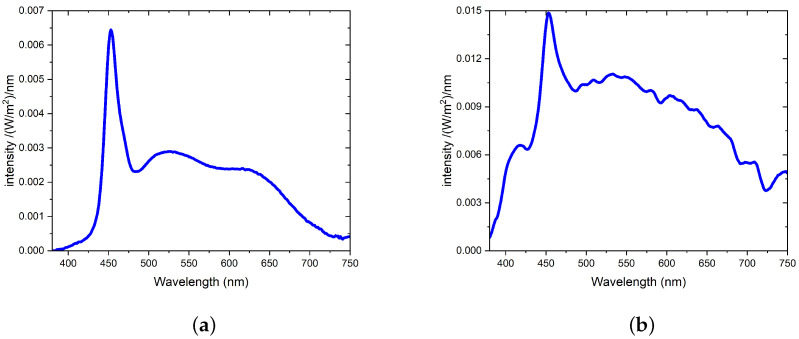
Representative spectra of point measurement and data logging results in patient room 9. (**a**) Spectrum of point 3 in patient room 9, taken at 10:08:45 a.m. on 12 January 2022. (**b**) Spectrum from data logging results in patient room 9, taken at 10:00:10 a.m. on 24 August 2022.

The results for point measurement, including sensor orientation, measurement time, measurement height, and illuminance, as well as the CCT, CRI, MEDI, and MDER values, are shown below in Table 2. A representative spectrum of point 3, taken at 10:08:45 a.m. on 12 January 2022, is shown in Figure 2a.

As shown by the spectrum in Figure 2a, on a winter morning with an overcast sky with the curtains drawn, the artificial lighting from the LED luminaires was the dominant light source in the room.

For the point measurement results, the illuminance values at all points provide a good vertical illumination at eye level. However, we have not assessed the effectiveness of the lighting in the context of the performance of visual tasks. For MEDI, the recommended value is above 250; the results for points 2, 3, and 5 were too low to provide circadian entrainment. The CRI values for all points were above 90, indicating a good color-rendering ability from the lighting system. By comparing the results, it can be seen that even and the curtains drawn, daylight still contributed to the results, as the west-facing (window-facing) measurement points show higher illuminance and MEDI values than the east-facing values, which is crucial in terms of the MEDI values meeting the minimum requirements.

### 2.4. Data Logging

For the data logging results, a representative spectrum taken at 10:00:10 a.m. on 24 August 2022 is shown in Figure 2b.

**Figure 3:**
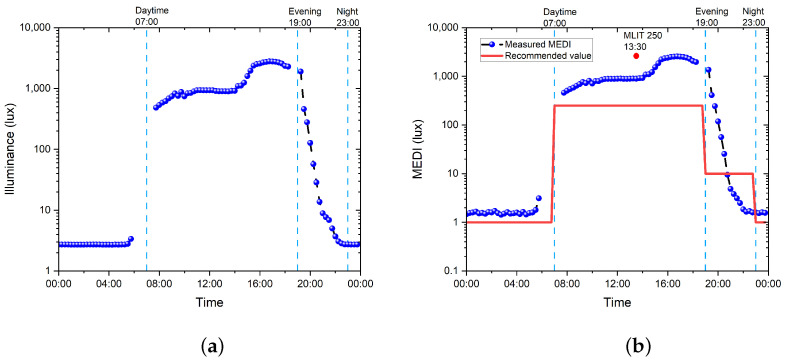
The 24 h progression of illuminance and MEDI values in patient room 9. (**a**) Progression of illuminance value; 07:00, 19:00, and 23:00 correspond to the beginning of daytime, evening, and night-time. (**b**) Progression of MEDI value; 07:00, 19:00, and 23:00 correspond to the beginning of daytime, evening, and night-time, separated with light-blue dashed lines, with the blue curve representing the measured data, the red line representing the recommended threshold by Brown et al. 2020 [22] and GLG 2023 [36], and the MLIT 250 representing the mean light timing above 250 MEDI, which was calculated at 13:30.

The 24 h progression of illuminance and the MEDI values in patient room 9 are plotted and presented in Figure 3a,b, respectively. Where 07:00, 19:00, and 23:00 correspond to the beginning of daytime, evening, and night-time, and are shown as dashed vertical lines. In Figure 3b, the red line represents the recommended threshold (<30 years) by Brown et al. 2020 [22] and GLG 2023 [36]. By using (1), the MLIT 250 (representing the mean light timing above 250 MEDI) was calculated at 13:30, as shown by the red dot, while the TAT during the day was calculated at 11.5 h.

**Figure 4:**
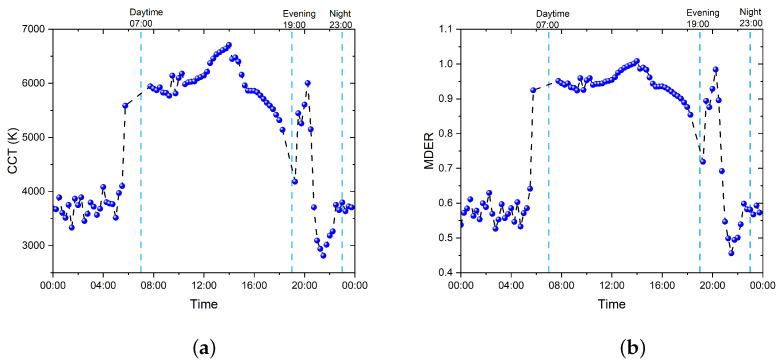
The 24 h progression of the MDER and CCT values in patient room 9. (**a**) Progression of the measured CCT values; 07:00, 19:00, and 23:00 correspond to the beginning of daytime, evening, and night-time, separated with light-blue dashed lines. (**b**) Progression of the calculated MDER (MEDI value divided by the illuminance value); 07:00, 19:00, and 23:00 correspond to the beginning of daytime, evening, and night-time.

The 24 h progression of the CCT values in patient room 9 are plotted and presented in Figure 4a. It can be seen that as the daylight coming through the window becomes the dominant light source after 08:00, the CCT values increased drastically to over 5000 K compared to the night-time, and this fits the typical sunlight range of 5000–6500 K. After sunset, the CCT values dropped to 2700–4000 K during evening and night-time, indicating that artificial lighting became the dominant light source in the room and from outside.

For the measured illuminance and MEDI values, the corresponding MDER values were calculated as the ratio between the MEDI and illuminance values; its progression over 24 h can be seen in Figure 4b. It is shown that, as the daylight is coming through the window and becoming the dominant light source after 08:00, the MDER values increased to over 0.9, indicating that the nonvisual light during the daytime had a very high melanopic content and can be characterized as stimulating light. After sunset, the MDER values dropped to 0.45–0.6 during evening and night-time, indicating that the dominant light source became the artificial lighting in the room; from outside, however, the value was higher than 0.3, meaning it cannot be characterized as a sleep-promoting lighting condition for the occupants in the room.

It should be noted that the Y-axis in Figure 3a,b are in a logarithmic scale, and the abnormal data points from 06:00 to 07:30 and from 18:30 to 19:00 were not included in the results. For those points found from 06:00 to 07:30, sunlight was coming through the blue curtain with the sunrise, and because the artificial lighting was off, sunlight was the main light source. The sensor was receiving blue light as a result, giving out abnormal CCT and MDER values. After 07:30, the curtains were open, resulting in normal measurements from the spectrometer. As for the points from 18:30 to 19:00, the sunlight hit the sensor directly through the window during sunset, resulting in abnormal illuminance and MEDI values (over 5000 lux). Thus, those data points were left out from the plots, and further calculations are needed to provide a more accurate analysis.

In future studies and measurements, it is important to avoid sunlight coming through a colored curtain or impinging directly onto the sensor since it can cause corrupted measurement results. In that case, outlier data should be spotted and removed in order to provide a more accurate calculation and analysis.

For the data logging measurement, it is shown that under optimal weather conditions with abundant daylight, the patient room equipped with an LED circadian lighting system has a high level of illuminance and MEDI for both illumination and circadian entrainment. The light level dropped significantly after 7:30 p.m., indicating that the light was dimmed down automatically and turned off after 10 p.m. (with the curtains closed at the same time). When comparing this with the point measurement, the contribution from the daylight has obviously boosted both the illuminance and MEDI levels, indicating that access to daylight is essential for both illumination and circadian entrainment.

From the results presented above, it can be seen that with the combination of on-site measurements from the gigahertz spectrometer and the calculations using the Luox platform, the light level of an indoor environment can be determined accurately. By using the point-measurement scheme, the light level at various positions and heights in the room can be measured, which, in turn, can map out the lighting distribution, including the contributions from both artificial and natural lighting in the room. By using data logging, the light history, zeitgeber strength, and temporal dynamics of the chosen location in the room can be determined. It should be noted that the MEDI requirements used in the plot and discussion are for occupants under 30 years of age; it should be adjusted based on the actual age range of the occupants when applied.

As can be observed in Figure 4a,b, there is a high correlation between CCT and MDER, whereas MDER is poorly correlated with MEDI in the evening and night-time data logging measurements. This indicates that MDER is an ambiguous predictor since MDER is significantly above the “sleep-promoting” threshold, i.e., MDER = 0.3, while the MEDI values are low. This also indicates that MDER is only valid for bright enough illumination conditions since it is defined as the ratio of MEDI over the photopic illuminance, as shown in Equation (2) below.

(2) $$MDER=\frac{\frac{1}{K_{mel,V}^{D65}}\int_{vis}^{}S_{mel}\left(\lambda \right)\cdot E_{e,\lambda }\left(\lambda \right)d\lambda }{E_{v}}$$

**Figure 5:**
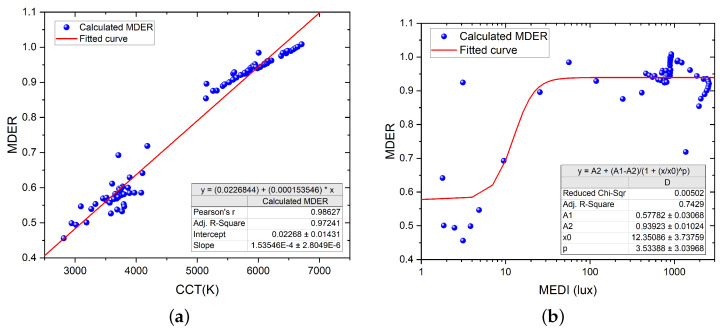
Regression between MDER and CCT, as well as between MDER and MEDI. (**a**) Linear regression between MDER and CCT. (**b**) Logistic regression between MDER and MEDI; the night-time values have been removed.

$K_{mel,V}^{D65}$ is equal to 1.3262 mW/lm for melanopic radiation, $S_{mel}$ is the melanopic action spectrum, $E_{e,\lambda }$ is the SPD of the light source of interest, and $E_{v}$ is the photopic illuminance. The numerator of the above fraction is the definition of MEDI, with the integration performed over the visible range. We refer the reader to the CIE S026 document for more details about these quantities [23]. We fitted the calculated MDER values to both the measured CCT and MEDI. We used a logistic regression for fitting MDER to MEDI since we assumed a nonlinear relation between these quantities given Equation (2). The MEDI and MDER values at night-time were removed to perform the regression. The results of both regressions can be seen in Figure 5. The $R^{2}=0.97$ for the linear fit between MDER and CCT suggests a strong correlation between the two metrics, which might, in turn, hint at a relationship between CCT and circadian entrainment. However, this linear correlation might only hold under specific conditions. Mathematically, Equation (2) defines the “closeness” of the spectral photopic radiation energy in lux for a target light source and relates this to the spectral melanopic radiation energy of a CIE D65 illuminant in lux. The CIE D65 illuminant approximates the spectrum of daylight at 6500 K. Since daylight can be considered as an approximated blackbody radiator, we, therefore, hypothesize that CCT can be a proxy for circadian changes for light sources that can be approximated by a blackbody radiator, i.e., daylight, a mix of LEDs and daylight, and spectrum-enriched LEDs (e.g., sun-like spectrum LEDs). However, this hypothesis needs further analysis in order to be fully endorsed.

## 3. Melatonin Suppression

The CIE S026 metrology system defines the action spectra of the five known photo-receptors of the human retina: the three types of cones and the rods, constituting the IF, i.e., visual system, and the ipRGCs, which are mainly responsible for the NIF responses [39]. The cones are divided in short (S), middle (M) and long (L), respectively, which are sensitive to short (peaking around 420–440 nm, medium (peaking around 530–540 nm), and long wavelengths (peaking around 560–580 nm). Melanopsin has been demonstrated to be the primary and strongest driver for circadian changes in humans [40,41,42], which makes MEDI the International system of unit (SI) metric of choice for assessing and designing integrative lighting schemes.

In addition to the specification of the action spectra for the five types of photo-receptors, CIE S026 provides a comprehensive calculation method to derive α-opic irradiances for each of the photo-receptors. However, quantifying the α-opic irradiances does not afford a direct method for assessing the exact effect of a photo-receptor on human health. To date, the most relevant methods to relate action spectra and their respective irradiance to potential light-induced health effects are dose-response curves, i.e., melatonin suppression and circadian phase resetting. From the work by Brainard et al. [43], the following melatonin suppression dose-response curve can be derived by fitting the four parameters’ sigmoid, as depicted in Equation (3).

(3) $$y=c+\frac{a-c}{1+{\left(\frac{x}{b}\right)}^{d}}$$

In the equation above, *a* and *c* are the lowest and the highest melatonin suppression levels, respectively, *y* is the response, *x* is the irradiance of the light stimuli, *b* is the stimulation value for which the response is half of its maximum value, and *d* controls the slope of the curve. The melatonin dose response outputs the percentage of melatonin suppressed by the action of a given light stimulation, depending on its strength and spectral composition. As discussed above, melanopic-irradiance is the strongest and primary factor responsible for melatonin suppression under a wide range of circumstances [41], especially for exposure longer than 90 min. However, the non-negligible contribution from the S-cone in early light exposure and at low light levels has recently been demonstrated [42,44,45], contradicting the findings from an earlier study [46].

Prior to the formalization of the S026 in 2018, a popular multi-opsins model for quantifying the action of light on the circadian system was developed by Rea et al. [47,48]. This model, which is termed the circadian stimulus model (CS) and is based on a dose response similar to melatonin suppression, defines a metric and uses the authors’ circadian light (CLA) as an input. The CLA is calculated using a complex multi-opsins photo-transduction mathematical model, accounting for the spectrally opposing signals and interactions of ipRGCs and the visual photo-receptors, i.e., cones and rods. Despite its elegant mathematical formulation and ease of use, because it outputs a single factor, i.e., CS, that is directly related to the action of light on health, and due to its popularity, the CS model remains controversial [27,28]; therefore, we did not include CS in our assessments. The CS is calculated using the equation below, in which CLA accounts for (circadian) light stimulation.

(4) $$CS=0.7-\frac{0.7}{1+{\left(\frac{CLA}{355.7}\right)}^{1.1026}}$$

The CS value bears some similarities to nocturnal melatonin suppression models, with a maximum suppression of 70%. However, according to Rea’s response to Schlangen [27] the CS model is not meant to be a melatonin suppression model. We refer the interested reader to the original papers from Rea et al. [47,48] and the detailed analysis of this model by Khanh et al. [49] for in-depth details and insights regarding the math and theoretical foundations behind the CS and CLA. The model has been improved to take exposure time and field of view into account. The modified equation is not shown here, but the details can be found in [49].

In order to investigate the usefulness of nocturnal melatonin suppression models from the literature as a baseline for assessing the circadian-related changes of a given lighting scheme in built-in environments, especially with regard to mental, neurological, and psychiatric health, we have processed our log over time data by using two recently published dose response models for melatonin suppression. The models are detailed in the remainder of this section. Since nocturnal melatonin suppression studies are conducted with a specific experimental setup that varies from one study to another, the obtained models might, therefore, not hold for other settings, in particular, extrapolation to real cases.

### 3.1. Prayag et al.

In a 2019 study [40], Prayag and colleagues derived dose responses for all five α-opic irradiances, as well as photopic illuminance, and found that melanopsin was the main driver for melatonin suppression. In their study, they specified the irradiances by using the system from Lucas et al. [21], which is closely related to the newly developed system from CIE S026, and the illuminance values obtained by Lucas et al. [21] can be converted into EDI lux. Their findings were later confirmed by Brown [41]. They first fit a four-parameter sigmoid to data from Brainard et al. [43] and then to the data obtained experimentally in their laboratory. The light exposure duration was 1 h in their study; the pupils of the participants were pharmacologically dilated, and plasma melatonin was sampled every 15 min from 30 min before and after light exposure (00:30–01:30 h).

Since they provided parameter fittings for their equation, we have decided to investigate the usage of this as a metric to assess the melatonin suppression effectiveness of the measured MEDI data logging. The maximum melatonin suppression for melanopic illuminance for their model is around 73%.

It should be noted that the authors of the study have discussed the limitations of their model regarding its scope in the discussion section of their paper, and they concluded that it might not be possible to generalize it to real-life conditions, as well as situations involving polychromatic lights or LEDs, and they insisted on factors that can impact the sensitivity thresholds, such as age, light history, etc.

### 3.2. Giménez et al.

Giménez et al. conducted a meta-analysis based on 29 melatonin suppression studies from the literature by using a random forest model to identify the key parameters for melatonin suppression [42]. They then fitted the gathered data by using a four-parameter sigmoid, similar to Equation (3). They derived a model that takes light exposure duration into account, as well as the dilated or nondilated status of pupils, since some studies have been conducted with and without pharmacologically dilated pupils. They allowed their model to reach 100% melatonin suppression, which aligns with observations from [27]. St Hilaire et al. [45] also used a high maximum melatonin suppression value for fitting their model, i.e., 95%, in their 2022 study. They thoroughly analyzed the influence of every single opsin and combination thereof by using the metrics from CIE S026 to assess the factors contributing the most significantly to melatonin suppression. It was found that MEDI was the most significant predictor of melatonin suppression, especially for longer and brighter exposures. However, the combination of both ipRGCs and S-cones resulted in a slightly better fit than ipRGCs alone, which indicates a non-negligible contribution of the S-cone to melatonin suppression, as shown in Brown et al. [44] and St Hilaire et al. [45]. However, they did not include the action of S-cones in the final model. Their findings also suggested that the S-cone was the best predictor at lower illuminance, i.e., under 23 lux.

The model from Giménez et al. [42], which can be viewed as an attempt to provide an SI compliant-based metric, an alternative to the CS model, by using the EDI from the CIE S026 [49,50], is described by the following equation:

(5) $$Melatonin_{suppression}=\frac{0-100}{1+{\left(\frac{log_{10}\left(EDI_{melanopic}\cdot 10^{6}\right)}{9.002-0.008\cdot \delta t_{exposure}-0.462\cdot dil_{pupil}}\right)}^{7.496}}+100$$

In the equation above, $\delta t_{exposure}$ is the exposure time to light stimulation in minutes, $EDI_{melanopic}$ is the MEDI, as defined by the CIE S026, $dil_{pupil}$ is the status of pupil dilation and is equal to 0 for a non-pharmacologically dilated (i.e., undilated) pupil and 1 for a pharmacologically dilated pupil.

### 3.3. Numerical Evaluation of the Models

Since Brown et al. [44], Giménez et al. [42], and St Hilaire et al. [45] have recently shown that the S-cone contributes to melatonin suppression at low illuminance levels, and in the early stage of light exposure, we have computed both the S-cone- and melanopsin-induced melatonin suppression over time using the equation of Prayag et al., melatonin suppression was calculated for every data point in data logging. We stress here that the results obtained by using the Prayag et al. model are only indicative and should only be cautiously interpreted due to the inherent limitations of the model, emphasized by the authors. However, it seemed interesting to us to investigate the extrapolation of such a model to real cases since it is based on a more closely related metric system than S026.

**Figure 6:**
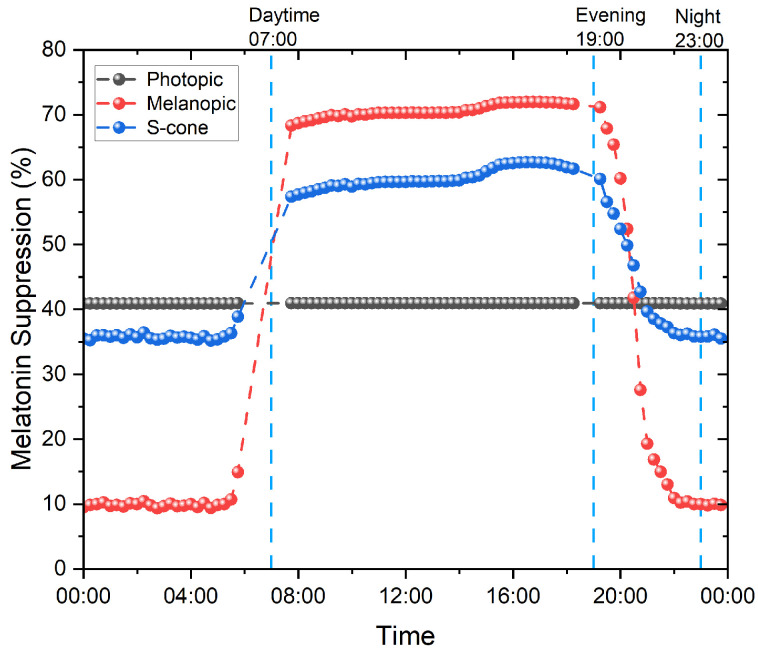
The 24 h progression of melatonin suppression using the Prayag et al. model for photopic, S-opic, and melanopic illuminances.

As can be seen in Figure 6, melatonin is predominantly suppressed by melanopsin during the daytime, reaching its saturation point, and during the early evening, where the light levels are high; for the S-cone during the night, early morning, and late evening, the light is very dim. These results align with the findings from Giménez et al. [42] regarding the contribution of the S-cone at lower illuminance levels. In Prayag et al. [40], the S-cone illuminance-response curve was found to be the third-best predictor of melatonin suppression ($R^{2}$ = 0.52). However, due to its average goodness of fit and the large variance in the fit parameters, the numerical values from the S-cone illuminance-response fit should be taken with caution. Furthermore, the fitted value for the maximum response from the S-cone varies between 92.64% ± 100.21, which is not interpretable from a physiological point of view.

The photopic illuminance elicits a sustained and constant suppression, regardless of the light levels, of around 40.96%, which corresponds to its saturation level. The reason for this is that the half saturation illuminance value for the photopic model reported in the original paper from Prayag et al. is equivalent to 0.19 lux. The M- and L-cones, for which the combined sensitivities roughly correspond to the photopic sensitivity function V(λ), were also found to saturate around 57.46% and 44.92%, respectively, with a very low half saturation of 5.29 and 1.27 lux, respectively, in the Prayag et al. study. The values for both maximum melatonin suppression and half saturation illuminance, as reported by Prayag et al. for the photopic system (L-cones, M-cones, and photopic), seem very low [40].

**Figure 7:**
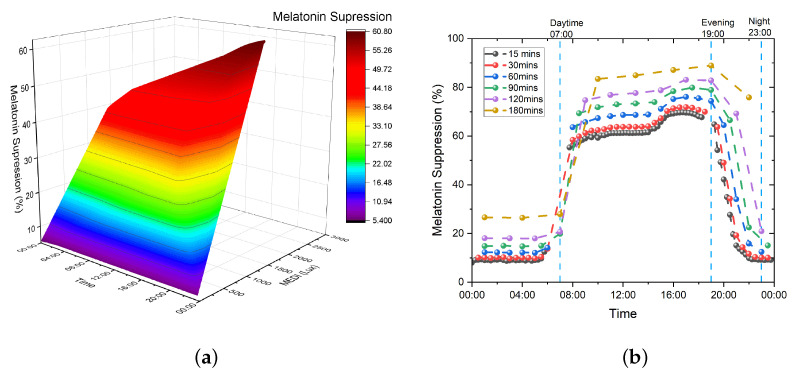
The 24 h progression of the melatonin suppression value in patient room 9. (**a**) Progression of melatonin suppression, considering 15 min of exposure. (**b**) Progression of melatonin suppression for all considered exposure times.

We then used the Giménez data on our measured data logging and used different exposure times, i.e., 15, 30, 60, 90, 120, and 180 min. Since light was measured every 15 min in our data logging, the calculated melatonin suppression values correspond exactly to the measurement points for the 15 min $\delta t_{exposure}$. For the other time exposure durations, the average MEDI over the given time period was calculated and used as an input to the Giménez et al. equation. Exposure time seems an important parameter to take into account since the different opsins follow a specific temporal dynamic [1,42,44,45], with melanopic-induced melatonin suppression being maximal from 90 min of exposure onward [1,45]. In our calculations, $dil_{pupil}$ has been set to 1 since we considered pharmacologically dilated pupils in our setting to ensure a fair comparison with Prayag et al., which was derived using pharmacological pupil dilation. The results for the data logging points, i.e., time exposure of 15 min, are depicted in Figure 7a below. The results for all exposure durations are depicted in Figure 7b.

When compared to the progression obtained using the work of Prayag et al. [40], the principal difference of the results by Giménez et al. [42] is that they do not saturate; melatonin suppression follows the same dynamic as the measured melanopic illuminance, regardless of the exposure time used in the equation. By using an exposure time of 12 h (720 min) at a sustained stimulation of 250 MEDI and the equation from Giménez et al., an optimal melatonin suppression of 99.85% can be obtained. This confirms the validity of the recommendation from Brown et al. and the GLG for daytime exposure, assuming that near-optimal melatonin suppression correlates with high positive cognitive and circadian changes in humans.

Regarding 60 min of exposure time, we compared Giménez et al. and Prayag et al. to match the experimental light exposure used by Prayag et al. We use the dilated pupil version of the Giménez et al. equation since that of Prayag et al. was derived from dilated pupils.

**Figure 8:**
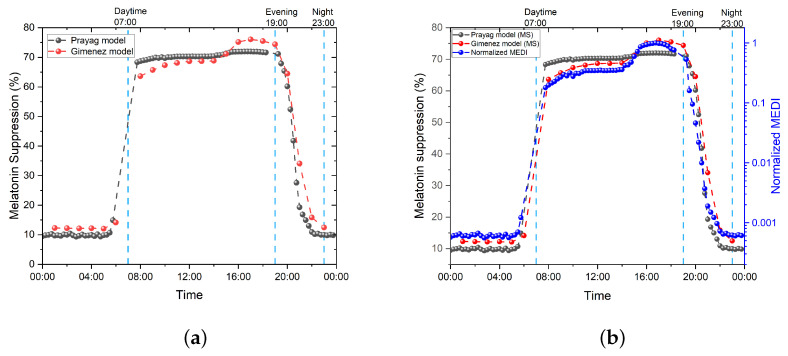
The 24 h progression of melatonin suppression for Prayag et al. and Giménez et al. (**a**) Progression of melatonin suppression values. (**b**) Progression of melatonin suppression vs. normalized MEDI data logging values.

The results shown in Figure 8 were obtained for every data logging point.

In Figure 8, we can see that the Prayag et al. version saturates at around 70–73%, while the Gimenez et al. version follows the exact same dynamic as the MEDI measurements. The accordance between the dynamic of melatonin suppression calculated with the Giménez et al. version, with a dilated pupil, 60 min of exposure time, and the progression of the MEDI values, can clearly be seen in the right graph Figure 8b. Again, we stress here that our measurement setup differs from the experimental conditions of Prayag et al. In addition, Prayag et al. used a single, albeit large, dataset, whereas the Giménez et al. version was derived using 29 datasets from the literature. Therefore, the limitations of the Prayag et al. version on our measurements do not conclude anything about the intrinsic quality of their model. We simply confirm here, as discussed by the authors, that the model is not meant to be a generic model and has not been designed to be a universal metric for the design and assessment of lighting installations.

**Figure 9:**
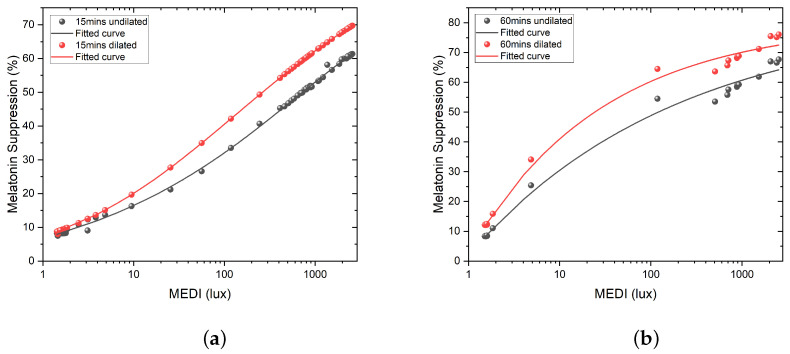
(**a**) Melatonin suppression for different MEDI exposure levels under a 15 min light exposure time, with dilated and undilated pupil versions of the Giménez et al. equation. (**b**) Melatonin suppression for different MEDI exposure levels under a 60 min light exposure time, with dilated and undilated pupil versions of the Giménez et al. equation.

The impact of pupil dilation can be seen in Figure 9 below, where we have compared the melatonin suppression for both the undilated and dilated pupil versions of the Giménez et al. equation [42]. It can be seen that the dilated version of the equation yields higher melatonin suppression levels since the retinal illuminance is higher when the pupil is dilated.

The effect of time exposure can also be seen; the dose response adopts a more pronounced logarithmic sensitivity as the duration of exposure increases. In addition to the shape of the response curve, a longer exposure time seems to also increase the discrepancy between the melatonin suppression values with or without dilation. Nie et al. [51] studied the effect of pupil dilation, the combination of opsins, exposure duration, and retinal illuminance in a similar approach to that of Giménez et al.; they conducted a meta-analysis of prior melatonin suppression studies as an attempt to derive a retinal-illuminance- and multi-opsins-based melatonin suppression model.

The results shown in Figure 9 emphasize the importance of pupil size for assessing the circadian response of a given light stimulation since the differences in melatonin responses are significant depending on the choice of the $dil_{pupil}$ parameter. Therefore, the choice of undilated and dilated pupil versions of the Giménez et al. model can be critical. In its current form, i.e., the published version of the model in [42], this model does not account for variations in pupil size, pharmacological dilation (in the source studies), and related retinal illuminance. A rule of thumb could be to empirically use the undilated pupil version for field studies in which it is assumed that pupils are freely allowed to change, although this approach might not be robust. Pupil diameter varies depending on multiple factors, including age, environmental luminance, field of view, the spectral composition of the stimulation, timing, etc. [52,53,54,55,56,57,58], and this accounts for the adaptation state of the visual system. Here, we can see a caveat of the model proposed by Giménez et al. [42] since its application to real-world cases might be complicated regarding which version of the model is relevant. Moreover, retinal illuminance is probably a better predictor of light-induced circadian changes than eye-level vertical illuminance, i.e., corneal illuminance, since the effective illumination reaching the retina depends on the optics of the eye, which includes pupil size.

**Figure 10:**
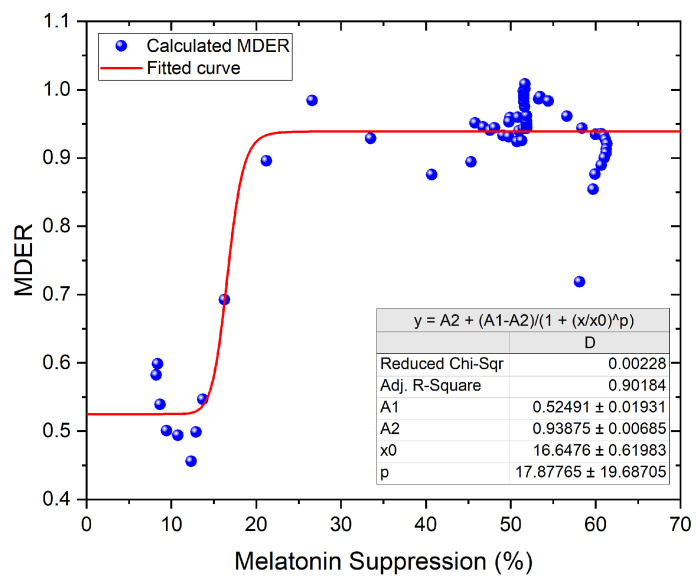
Logistic regression between MDER and melatonin suppression using the Giménez et al. model, with night-time values removed, pupil dilation set to 0, and exposure time set to 15 min.

In order to assess the relation between MDER and melatonin suppression, we performed a logistic regression between the two metrics, as shown in Figure 10 below. Here, we used a 15 min exposure time to match the sampling rate of our data logging measurements, with the night-time measurements removed, and set the pupil dilation to zero. The $R^{2}=0.90$ indicates a relatively good correlation between the two metrics. However, since MDER does not seem to be a good predictor at low illuminance, a melatonin suppression model able to investigate S-cone-induced melatonin suppression at lower EDI values could help to better understand the effects of light in the evening and at night, assuming that the recent findings from Giménez et al. are confirmed by other studies.

## 4. Discussion

### 4.1. Discussion of the Results

The study detailed in this article was meant to define a field measurement protocol and its dissemination in the context of evaluating the circadian and photometric performance of a given lighting scheme in built environments. In the specific case presented here, we have defined a methodological approach suited for hospital rooms with patients suffering from neurological, psychiatric, or mental disorders. We also have reviewed the current state of knowledge of circadian lighting and integrative lighting research and best practices, hoping to inspire human-centric lighting practitioners.

For the point measurement results, the illuminance values at all points provide good vertical illumination at eye level. However, we have not assessed the effectiveness of the lighting in the context of performing visual tasks. By comparing the results, it can be seen that even with the curtains drawn, daylight still contributed to the results, as the west-facing (window-facing) measurement points show higher illuminance and MEDI values than those of the east-facing condition, which is crucial for the MEDI values to meet the minimum requirements.

For the data logging measurement, it is shown that under optimal weather conditions with abundant daylight, the patient room equipped with an LED circadian lighting system has a high level of illuminance and MEDI for illumination and circadian entrainment. When compared with the point measurement, the contribution from daylight has obviously boosted both the illuminance and MEDI levels, indicating that access to daylight is essential for both illumination and circadian entrainment. However, the lighting conditions at night were not ideal for sleeping at night, as both the MEDI and MDER values were too high for sleep promotion; thus, it is important to dim the light in the room before sleep and to block stray light from outside.

From the results presented above, it can be seen that with the combination of the on-site measurement using a gigahertz spectrometer and the calculations using the Luox platform, the level of light in an indoor environment can be determined accurately. By using the point-measurement scheme, the light level at various positions and heights in the room can be measured, which, in turn, can map out the lighting distribution, including the contributions from both artificial and natural lighting in the room. By using data logging, the light history, zeitgeber strength, and temporal dynamics of a chosen location in a room can be determined. In order to perform more accurate calculations and analyses in future studies and measurements, it is important to avoid sunlight coming through a colored curtain or incident directly onto the sensor to disrupt the measurement results, and outliers should be spotted and removed.

### 4.2. Perspectives and Future Work

In our assessment, we have also included nocturnal melatonin models in order to understand their applicability in such field studies. In many real-world light engineering studies [59,60,61,62], the CS model has been used as a metric for evaluating the circadian performance of a given lighting scheme. We did not include the CS in any of our field studies since the validity of CS is still being debated. On the other hand, the SI metrological system developed by the CIE provides a universal, standardized, and robust framework for quantifying the biological responses of retinal photo-receptors to light. However, to date, there is no S026-based model equivalent to the CS model. The Giménez et al. model is an interesting approach for a MEDI-based circadian metric geared towards light engineering. The two models have been compared in [49,50]. Khank et al. also found a high correlation between the models and, therefore, modeled a mathematical linear relationship between CLA and MEDI, allowing the conversion of CLA into MEDI. We have used the Giménez model and found that it can be used in this context, providing that the pupil dilation and time exposure parameters are correctly understood. However, the Giménez model has some restrictions, as discussed by the authors. First of all, the impact of previous light history is not modeled despite modeling exposure duration. It is also unclear how to include the dynamics of pupil variation and adaption to light in that framework. Zandi et al. [56] have proposed an interesting time and spectral-dependent pupil model, extending Watson’s work. Moreover, Gooley et al. [1], Brown et al. [44], and St Hilaire et al. [45] have demonstrated that melatonin suppression was not only dependent on melanopsin; it was dependent on the dynamic contribution of both ipRGCs and S-cones. Melatonin appears to be mainly suppressed by the action of the S-cones in the early exposure stages; then, the contribution of S-cones rapidly and linearly decays, finally vanishing after 90 min. From 90 min of exposure onward, ipRGCs are the only contributors to melatonin suppression. Giménez et al. have themselves shown that at lower illuminance, i.e., under 23 lux, the S-cones were the best predictor of melatonin suppression.

In light of these findings, a dynamic melanopsin/S-cone model could be considered for a better assessment of melatonin suppression in dim-light environments. If the Giménez model is adapted for static measurements, it is unclear how to properly use it in a dynamic context, for instance, with data logging. It would be of interest to investigate a dynamic version of the Giménez model that could be used in the context of real-time measurements. Another inherent limitation of such models is the variability of factors impacting melatonin suppression [63]. In the future, the in-depth assessment and validation of this model is needed, both mathematically and experimentally.

It seems to us that many of the limitations of the current practice for field measurements could be alleviated by using circadian light dosimeters [64,65,66]. However, to date, and to the best of our knowledge, there are no such devices available on the market, apart from the Lys button from Lys technologies [67]. However, this device is based on an RGB sensor and does not provide raw spectral data. Circadian Health Innovations [68], a spin-out from Monash University, has been working on miEye [69], a spectrally resolved circadian light logger. This device has not hit the market as of the time of writing, even though it was announced in 2021 that it would be ready for mass production [70]. Integrative-lighting-enabled actigraphs, like ActLumus [71], developed by Condor Instruments, can be an interesting alternative; however, as these are worn at the wrist level and are subjected to shading and occlusion if worn with long sleeves, they might be challenging to use in most real-life situations. NanoLambda (South Korea) has developed a nano spectrometer, XL-500 [72], which has a very small form factor and is Internet of Things (IoT)-enabled; this might also be considered in these kinds of studies. The device also supports the CIE S026 calculations. Unfortunately, its price is a limiting factor for deployment in large-scale studies involving dozens of participants.

As for the practical implications of our findings regarding any future designs of integrative lighting in hospital wards or other care facilities, we stress the importance of defining the MEDI lux based on retinal illumination rather than corneal illumination. This would allow researchers to take different age groups into account, as well as adaption, since pupil size varies according to the adaption of the visual system to the lit environment. In a dimly lit environment, the pupil is more likely to be larger and let more light in, impinging the retina and resulting in higher retinal illuminance. In a bright environment, the pupil will constrict and reduce the amount of light reaching the retina. Due to the wide variability of pupil size in younger individuals, i.e., from 2 mm in bright light to 8 mm in a dark environment, the variability in available retinal illumination is large. Thus, corneal illuminance might not be an accurate predictor. Moreover, as can be seen in Figure 9, the size of the pupil has an important impact on circadian changes. Since recent studies have pointed out the contribution of S-cones at lower light levels and during the first 90 min of light exposure, it seems that S-opic should be considered for the design of lighting schemes in the evening, especially for situations in which the patient needs to use light at night. For instance, caution might be taken to install light sources with lower energy in the 420–460 nm range of the visible spectrum. Such light sources can be installed in bathrooms, for instance, thus avoiding any circadian phase shift while using the restroom at night. In addition, light history can be used to better fine-tune the light levels in the evening since long and sustained bright light exposure will result in a higher threshold for the induction of light-related circadian changes. In hospital wards, patients and medical staff are more likely to be exposed to more or less constant illumination for a long period of time; therefore, light history is easier to evaluate in this context. These recommendations also enable a more individualized and targeted light design, addressing the needs of a specific population in terms of light exposure. Moreover, mitigating the risk of circadian imbalance for medical night shift staff is critical. Finally, spectrally optimized light sources with an enriched spectrum, i.e., providing an MDER closer to unity, should be preferred for entraining the circadian system during the day.

Our findings also emphasize the need for further research for the appreciation of the role of each photo-receptor and its light-induced nocturnal influence on physiological and psychological changes. It has been hypothesized in recent studies that S-opic EDI could be a better predictor of light-induced nocturnal melatonin suppression at lower light levels and during the first 90 min of light exposure. Confirming and extending these results would be of tremendous importance for future designs, as outlined above. We also have shown that melatonin suppression models could be used as predictors of the impact of a given light scheme on circadian changes in humans. However, the definition of a model incorporating S-opic EDI and its dynamics would be an important extension of the existing models. Such models should also include light history and retinal illuminance in order to provide a more accurate prediction of the circadian changes for targeted individuals and lit environments, thus allowing more precise fine-tuning of the illumination schemes of the lit space. Furthermore, it appears that the available metrics and models still need to be more thoroughly related to physiological and psychological changes in humans. To this end, longer field studies conducted with different populations and lighting conditions, in which both light indicators and physiological/psychological markers are collected over time, could help in understanding the exact relation of metrics scores and induced changes. In addition to melatonin, it could also be interesting to include measurements of cortisol and dopamine levels over time in long-term field studies at hospital wards. The role of cortisol in regulating the circadian system and its light-adjusted levels have been investigated [73,74,75,76]. Dopamine, which is a well-known regulator of retinal functions, especially adaption [77], has recently emerged as a candidate for regulation of the circadian system [78,79,80,81,82].

### 4.3. Limitations

Although a state-of-the-art, calibrated gigahertz spectrometer was used in this study, the instrument has some limitations at low light levels during the night due to noise; thus, the outlying data points during the night and early morning have been removed from further analysis, and such points should be noted in future studies.

In the present study, we used 175 cm as the highest level to simulate the eye height of medical staff (either a male nurse or doctor). Different heights should be considered in order to take into account both genders. It should also be noted that the MEDI requirements used in the plot and discussion are for occupants under 30 years of age; it should be adjusted based on the actual age range of the occupants when applied.

Another limiting factor is the sample size used in this study: one patient room with a west-facing window and only 24 h of data logging in summer. Since daylight varies in its nature, ideally, such field measurements should be conducted over longer periods, for instance, week-long data logging periods repeated throughout the four seasons, and use rooms of different orientations to account for variability in daylight for a given premise.

The assessment of S-cone contribution to melatonin suppression was conducted using the model from Prayag et al. [40]. However, we found that this model had some mathematical flaws. Therefore, experiments with a better S-cone model, for instance, that of Brown et al. [44] or Giménez et al. [42], should be considered in the future.

Lastly, the patient room in this study has the customized Chromaviso circadian LED lighting system installed, which adjusts the light level and spectrum automatically for circadian entrainment. However, this is not the case for most hospital wards; thus, it would be beneficial for future studies to include places with other light sources.

## 5. Materials and Methods

### 5.1. Materials and Equipment

**Figure 11:**
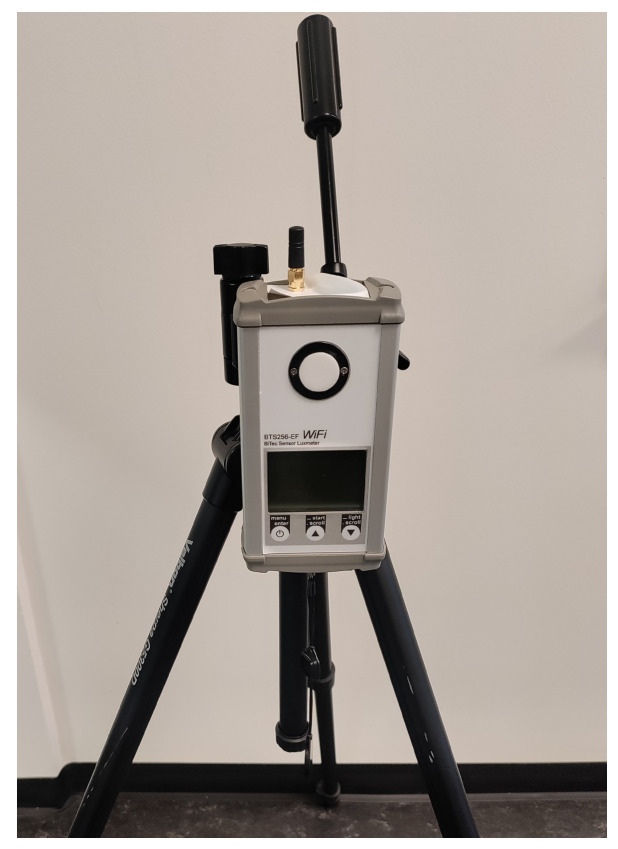
Measurement setup of the Gigahertz BTS256-EF Spectrometer on a tripod.

The equipment for our measurement schemes consisted of a BTS256-EF Spectrophotometer (Gigahertz Optik, Türkenfeld, Germany), a tripod with adjustable height and angle to mount the spectrophotometer, and a laptop installed with the S-BTS256 companion software version 2019.7 for point measurement, and 2022.3.0 for datalogging measurement, as shown in Figure 11 (laptop not shown). As the essential part of the measurement, the spectrophotometer covers the spectral range from 380 to 750 nm, capable of calculating α-opic EDI values in accordance with the CIE S026/E:2018 standard [23] and data logging using customized measurement intervals, which is suitable for our continuous measurement scheme over time.

### 5.2. Data Processing and Presenting the Results

For data processing, the measured spectra were exported into a *.CSV (comma-separated) file, then uploaded to the Luox platform [83] for the calculation of the relevant quantities (including illuminance, chromaticity,  α-opic irradiance, and α-opic daylight illuminance). The software used for the Luox calculation is endorsed by CIE, having made a black-box evaluation of the software as of 11 February 2021, finding that the software performs satisfactorily in calculating the quantities and indices derived from CIE publications [83,84].

Apart from the parameters mentioned above, the value of MLIT was calculated based on the measured MEDI values from the data logging results and the formula from Reid et al. [38]. Furthermore, with the 24 h logging of illuminance and α-opic daylight illuminance values, based on the models and parameters proposed by Giménez et al. [42] and Prayag et al. [40], we were able to calculate the melatonin suppression induced by S-cone and melanopsin over time, as well as the melatonin suppression response curves. This helped us to compare the two models and investigate the impact of exposure time and pupil dilation on the circadian response to light.

The calculated parameters were then exported and used in the measurement report represented the Supplementary Material. This report format is designed by our group using the online design platform Canva [85], allowing the input of each measured/calculated parameter, as well as the pictures of the light source, measurement setup, and spectral power distribution (SPD). Moreover, by utilizing the bars and speedometer design for data presentation, the measured results can be showcased in a more graphic and straightforward manner, making it easier to evaluate the light at a specific position from both visual and nonvisual aspects. All other calculations and statistics have been performed with Microsoft 365 Excel (Microsoft, Redmond, WA, USA) and OriginPro 2023 (OriginLab Corporation, Northampton, MA 01060, USA). OriginPro has also been used for the visualization and graphical presentations. No code has been generated.

This format was made using the Canva platform, and it includes pictures of the light source and measurement setup, showcasing the measured and calculated parameters.

### 5.3. Setup and Measurement Procedure of the Case Study

The point measurement was chosen to take place in the winter on the morning of 12 January 2022. With less daylight availability in the winter, an overcast sky on that particular day, and closed curtains, the daylight contribution to the overall lighting environment in the room was minimized; thus, the measurement was mainly focused on the performance of the LED lighting system, and the photometric, colorimetric, and nonvisual aspect of the lighting system was evaluated.

**Figure 12:**
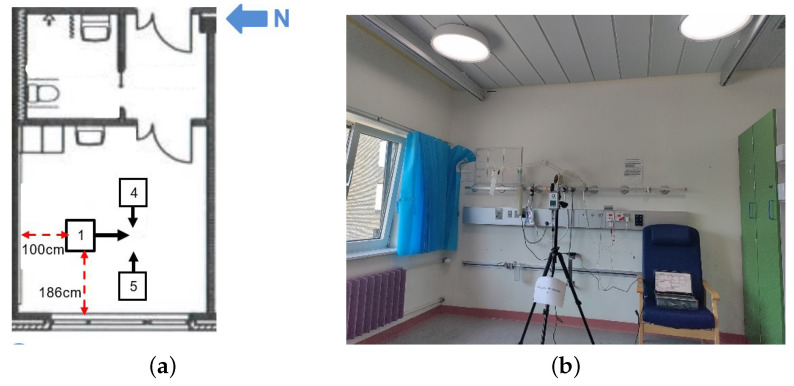
Picture of the measurement setup and floor plan with measurement points in patient room 9. (**a**) Floor plan of patient room 9, including measurement points, where points 1, 2, and 3 in the point measurement and the point in the data logging share the same spot. (**b**) Picture of data logging measurement setup.

The floor plan of the patient room, including the location of measurement points, can be seen in Figure 12a. The setups of the spectrophotometer, tripod, and laptop are identical for both point measurement and data logging, as shown in Figure 12b.

The point measurement was conducted according to the following setups:

1. Five measurement points have been chosen. Points 1 to 3 were chosen near the bed, imitating the patient-lying position in the bed facing a different direction;
2. Points 1 and 2 at 90 cm in height, facing west (window) and east, respectively, and point 3 at 104 cm in height, facing south;
3. Points 4 and 5 were chosen on each side of the bed at 175 cm in height, imitating the medical staff performing visual tasks in a standing position. Please note that this height would correspond to a male nurse or doctor, and therefore, a lower height should have been considered to assess the light exposure of a female nurse. However, the difference is too small to have a huge impact;
4. The spectrometer was mounted on a tripod facing the sensor, as indicated above, imitating eye height.

The data logging measurement was chosen in summer, between 23 and 24 August 2022. With abundant daylight through the window in summer, a sunny sky, and open curtains during the day (closed between 10:00 p.m. and 7:30 a.m. by hospital personnel), the daylight contribution to the lighting environment in the room was included; thus, the data logging was mainly focused on the overall lighting level in the patient room. Apart from the photometric, colorimetric, and nonvisual aspects of the lighting level, the contribution of daylight can also be observed by comparing it with point measurement, where the main light source was LED luminaires.

The measurement point was chosen to be the same as point 3, as shown in Figure 12a, with a measurement height of 140 cm, imitating a patient sitting in bed facing south. The measurement interval was set at 15 min, and the light level and spectrum of Chromaviso LED luminaires were adjusted automatically.

## 6. Conclusions

### 6.1. Summary of Results

The suggested method for measuring indoor integrated lighting environments includes point measurements and data logging. This method can represent lighting distribution, including contributions from both artificial and natural lighting. Additionally, it can indicate light history, zeitgeber strength, and the temporal dynamics of the chosen location in the room. The measurement scheme offers valuable information to researchers, designers, and other indoor professionals regarding the lighting situation in a given environment. This information can be used to improve lighting conditions and integrative lighting in terms of human mental and physical health, as well as human sleep and performance. Thus, this scheme is suitable for application regarding any measurements within indoor environments of integrative lighting performance and study.

The use of the Luox platform [83] ensured that the calculations of key parameters were accurate and aligned with the CIE requirements [23,84]. The template made using the Canva platform provides a straightforward and visual presentation for each measurement point, making it easier to get an overview of the light level from both visual and nonvisual perspectives.

It can be concluded that the measurement scheme consisting of point measurement and data logging, together with the use of the Luox platform [83] and the Canva template, is suitable for the accurate and satisfactory measurement of integrative lighting, aligning with CIE requirements and other widely accepted requirements [22,23,84] to provide a straightforward and graphic way of presenting the measurement results.

It has also be determined that, despite its limitations, the Giménez et al. model can be used as a baseline for a MEDI-based metric alternative to the CS model. However, based on our case study, the support of an S-cone melatonin suppression model is a much-needed feature for better understanding exposure time parameters and, ideally, a model that supports retinal illuminance, i.e., pupil size variation.

### 6.2. Significance and Outlook

In light of the work presented here, we have highlighted key factors for the evaluation of circadian performance in terms of lighting in built environments in field studies and, more specifically, in care facilities aimed at patients with poor mental health. We also determined and identified the approaches, models, and tools that seemed the most appropriate to us while also discussing their limitations. Our study has outlined the necessity for the usage of retinal illuminance instead of corneal illuminance for the prediction of the biological impact of a given light source, as well as the critical importance of more investigations for understanding the role of S-cones in light-induced circadian changes. Moreover, it has also been highlighted that temporal parameters, such as duration of exposure and light history, are necessary for an accurate assessment of the effect of illumination in terms of circadian changes. Ideally, data logging over longer periods and at different heights should be considered. However, traditional spectrometers are not suitable for these tasks, and spectrally resolved dosimeters appear to represent the best approach. The second author is currently developing a spectrally resolved and wearable circadian light logger that might be suitable for this purpose. In addition, the development of an S026-based metric alternative to the CS model seems a necessity. Giménez et al. paved the way toward such a model, and future work in our group will include work in that direction. Such a model should include retinal illuminance (hence, adaptation) and S-opic- and melanopic-driven melatonin suppression, and finally, it should be dynamic.

In our study, we found that MDER was not a suitable indicator under lower light levels, CCT might potentially be used as a proxy for circadian changes under certain lighting conditions, and finally, melatonin suppression and MEDI were correlated and were both excellent predictors of circadian changes. Furthermore, the exact relation of the photometric and circadian indicators with effective changes in human health is still yet to be fully understood. Our study has highlighted some of the limitations of these metrics in real-case applications. Accordingly, it is of critical importance that long-term field studies that investigate the relation between parameters, such as nocturnal melatonin suppression, MDER, CCT, MLIT, or MEDI, as metrics for the evaluation of light in a built environment (and the specific physiological and psychological changes in the occupants) are conducted in the future. Ideally, such studies should include different populations, i.e., different age groups, patient types, and staff categories (doctors, nurses, etc.), as well as different lighting conditions and types of light sources. It has also become apparent to us that the careful assessment of the available models and metrics should be conducted beforehand in order to evaluate their applicability and limitations in real-world applications. The model from Giménez et al. was studied in the context of our data logging measurements and was found to be very satisfactory, providing that the parameters of the equation are correctly understood and set appropriately. Improving this model to include the support of light history, the contribution of s-opic EDI, and retinal illuminance is a high priority and an absolute necessity for the integrative lighting community, including engineers, light designers, and sleep researchers.

In summary, we demonstrated that the existing metrics and models had some limitations in the real-world design and evaluation of circadian performance in terms of light schemes in a built environment, and extreme caution should be taken when using them. We have also stressed that a better understanding of the exact relation between the metric and model scores for circadian-related changes in humans was needed. We emphasized that temporal parameters are critical for assessing the performance of a given lighting scheme; these include duration of exposure, light history, adaptation (i.e., changes in pupil size and retinal illuminance), and photo-receptor dynamics and contribution to melatonin suppression. We then outlined the need for more research with regard to understanding the role of the S-cone in circadian changes and analyzing the relation of existing metrics and models to physiological and psychological changes. Finally, we recommend the development of a more suitable measuring device for field studies, which could include light dosimeters. We have found that despite its limitations, the model by Giménez et al. [42] is a very promising SI-based model and is a viable alternative to the circadian stimulus model.

## Figures and Tables

**Table 1:** Recommended minimal light levels on the eye for MEDI [36].

Recommended Minimal Light Levels on the Eye in MEDI	<30 Years	∼50 Years	>75 Years
**Daytime (7 a.m.–7 p.m.)**	MEDI ≥ 250 lux	MEDI ≥ 300 lux	MEDI ≥ 425 lux
**Evening (7 p.m.–11 p.m.)**	MEDI ≤ 10 lux	MEDI ≤ 12 lux	MEDI ≤ 17 lux
**Nighttime (11 p.m.–7 a.m.)**	MEDI ≤ 1 lux	MEDI ≤ 1 lux	MEDI ≤ 2 lux

**Table 2:** Measurement results for point measurement in Roskilde hospital patient room 9 (12 January 2022).

Measurement Point	1	2	3	4	5
**Orientation**	West	East	South	West	East
**Measurement time**	10:03:34 a.m.	10:04:31 a.m.	10:08:45 a.m.	10:14:22 a.m.	10:17:50 a.m.
**Measurement Height (cm)**	90	90	104	175	175
**Illuminance (lx)**	234.2	148	190.3	278.8	231.9
**CCT (K)**	7475	5848	6793	7924	6198
**CRI (Ra)**	96.4	96.3	96.4	96.1	96.1
**MEDI (lx)**	252.8	136.6	193.9	310.8	222.1
**MDER**	1.08	0.92	1.02	1.12	0.96

## Data Availability

The raw data obtained from Gigahertz spectrophotometer for both point and datalogging measurement can be found in Supplementary Material.

## Supplementary Materials

The following supporting information can be downloaded at: https://www.mdpi.com/article/10.3390/clockssleep5040052/s1.

## Abbreviations

The following abbreviations are used in this manuscript:

IF	Image-Forming
NIF	Non-Image-Forming
ipRGCs	Intrinsically Photo-sensitive Retinal Ganglion Cells
α-opic EDIs	α-opic Equivalent Daylight Illuminances
MEDI	Melanopic-EDI
GLG	Good Light Group
CIE	Commission Internationale de l’Éclairage
MDER	Melanopic Daylight Efficacy Ratio
CCT	Correlated Color Temperature
TAT	Time Above Threshold
MLIT	Mean Light Timing
SPD	Spectral Power Distribution
SI	International System of Units
CS	Circadian Stimulus
CLA	Circadian Light
