# Supplementary material for: Integrative Lighting Aimed at Patients with Psychiatric and Neurological Disorders
Source: Clocks Sleep. 2023 Dec 15;5(4):806–30. doi: 10.3390/clockssleep5040052 (PMC10742818; doi:10.3390/clockssleep5040052)
Supplement: Supplementary file 1 [file clockssleep-05-00052-s001.zip › Example of Canva measurement template.pdf]

Test: **Example for visual presentation of the results**

Measurement Location (1st floor, room type: patient room)

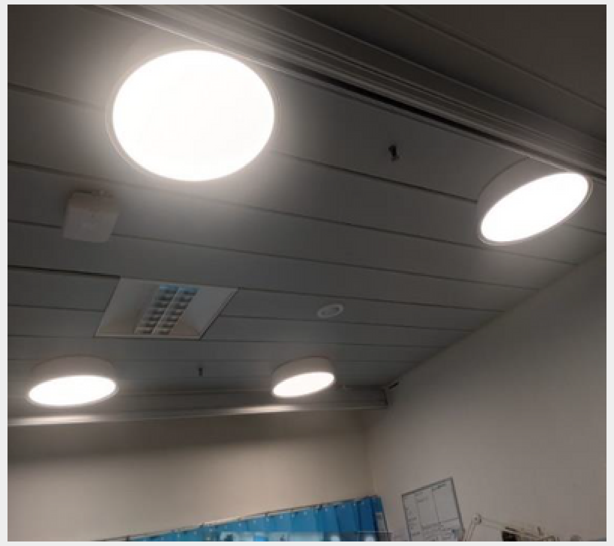

Measurement Point	3
Orientation	South
Measurement Time	10:08:45 AM 12-01-22
Measurement Height (cm)	104

Measurement of Spectrum Within the Visual Field (SPD)

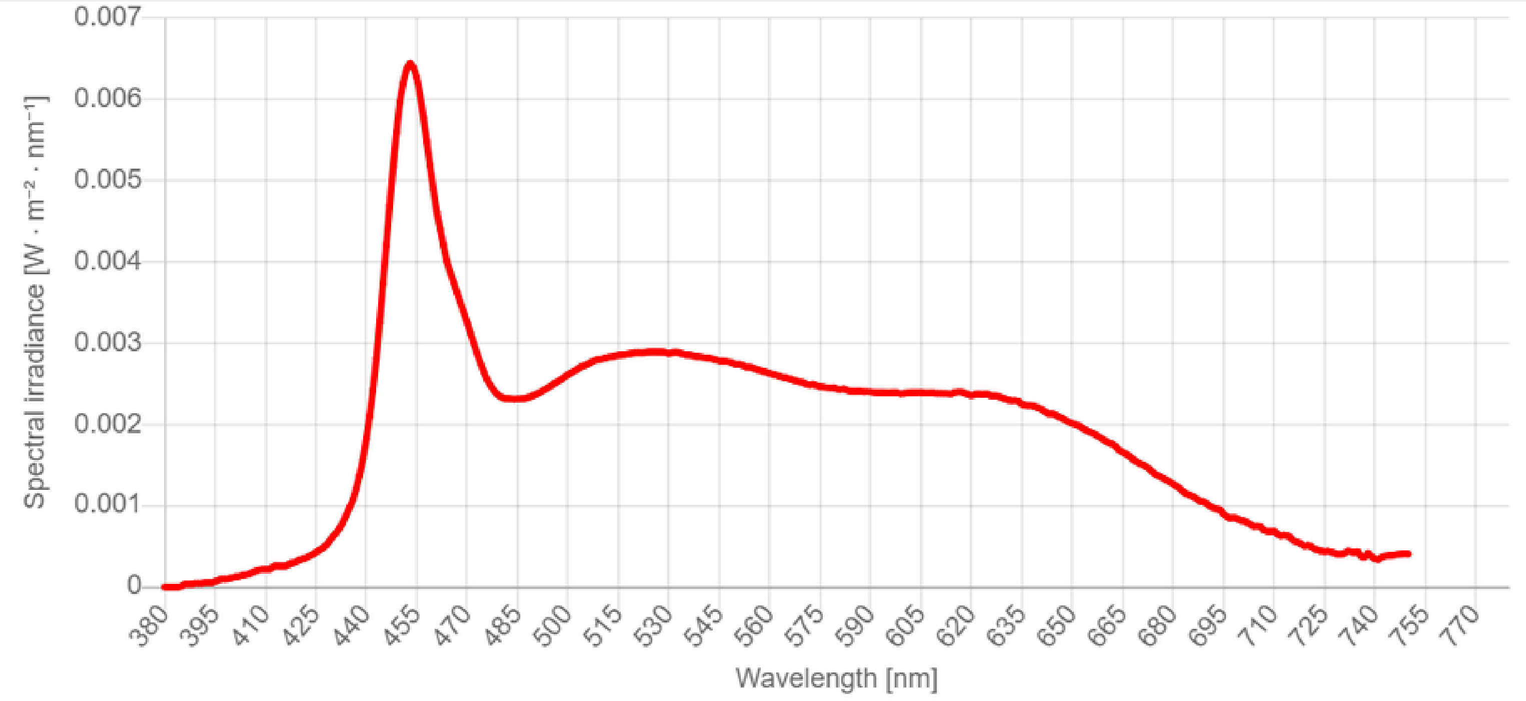

Visual (Image-forming Light)

Illuminance (Lux)

190

0 — 1000

Color-rendering (CRI)

Threshold

96

0 — 80 — 100

Colortemperature (K)

6793

2800 — 4000 — 6500 — 10.000

Non-image forming light (Integrative Lighting)

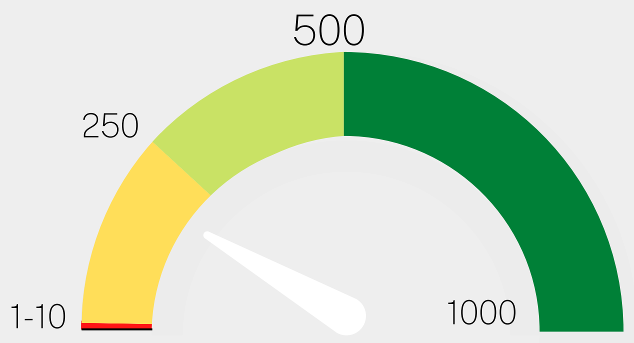

194

M-EDI (LUX)

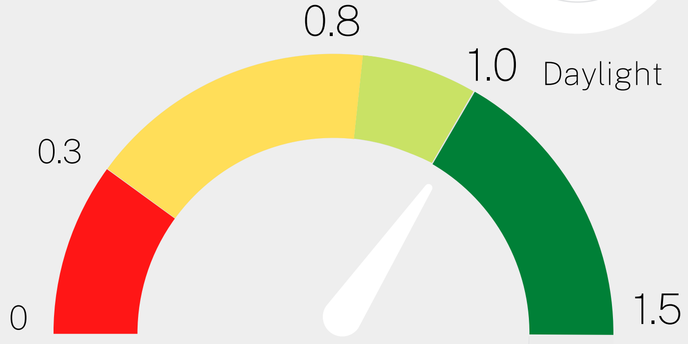

1.01

MDER

- Sleep promoting - 1 MEDI
- Active/ Awake Night - 10 MEDI
- Below Daytime Threshold
- Min. Daytime Threshold - 250 MEDI
- Superior Daytime Threshold - 500 MEDI

- Sleep Promoting - 0.3
- Active Light - 0.8
- D65 Daylight - 1.0
